# Description of *Sarcocystis platyrhynchosi* n. sp. (Apicomplexa: Sarcocystidae) from domestic ducks *Anas platyrhynchos* (Anseriformes: Anatidae) in China

**DOI:** 10.1186/s13071-023-05656-w

**Published:** 2023-02-02

**Authors:** Junjie Hu, Mingzhu Zhang, Zhipeng Wu, Hongxia Zeng, Jianping Tao

**Affiliations:** 1 School of Ecology and Environmental Sciences and Yunnan Key Laboratory for Plateau Mountain Ecology and Restoration of Degraded Environments, Yunnan University, Kunming, 650091 China; 2 College of Veterinary Medicine, Yangzhou University, Yangzhou, 225009 China

**Keywords:** *Anas platyrhynchos*, *Sarcocystis*, Ultrastructure, 18S rDNA, 28S rDNA, Mitochondrial *cox*1

## Abstract

**Background:**

Limited data are currently available on protozoan parasites of the genus *Sarcocystis* that infect their avian hosts within the order Anseriformes (waterfowl). To date, no *Sarcocystis* species has been recorded in ducks in China.

**Methods:**

Leg muscles were sampled from 26 domestic ducks (*Anas platyrhynchos*) in China in 2021. Morphological characteristics of sarcocysts detected in the muscle tissue were described using light microscopy (LM) and transmission electron microscopy (TEM). Genomic DNA was extracted from single sarcocysts obtained from different ducks, and three genetic markers, 18S ribosomal DNA (18S rDNA), 28S ribosomal DNA (28S rDNA) and mitochondrial (mt) cytochrome oxidase subunit 1 (*cox*1), were amplified and cloned for sequence analyses.

**Results:**

Sarcocysts were observed by LM in only three of the 28 samples (10.7%). These sarcocysts had a thick cyst wall with numerous brush-like villar protrusions (vps) of 3.8–4.3 μm in length (*n* = 30) on the cyst surface. TEM observation showed that the sarcocysts had lanceolated vps. Each vps narrowed in the stalk and contained a bundle of microtubules that extended into the ground substance. Comparisons of the new sequences with those deposited in GenBank showed that the most similar sequences were those of *Sarcocystis* *halieti* in the great cormorant *Phalacrocorax carbo* and European starling *Sturnus vulgaris*, and *Sarcocystis calchasi* in the domestic pigeon (*Columba livia*) at the 18S rDNA (99.1% identity); *Sarcocystis* *wenzeli* from the domestic chicken *Gallus gallus* at the 28S rDNA (95.9–96.0% identity); and *Sarcocystis speeri* from the opossum at the mt*cox*1 (98.2% identity). The new 18S rDNA, 28S rDNA and mitochondrial *cox*1 sequences shared up to 99.0%, 95.6% and 97.7% identity, respectively, with those of *Sarcocystis* spp. obtained from Anseriformes avian hosts. Phylogenetic analysis inferred from the sequences of the three genetic markers placed the organism within a group of *Sarcocystis* spp. obtained from avian or carnivorous intermediate hosts and avian, marsupial or carnivorous definitive hosts. Based on the morphological observation and molecular analyses, the organism found in the Chinese domestic ducks was regarded as a new species and named *Sarcocystis platyrhynchosi* n. sp.

**Conclusions:**

Based on morphology and sequence analyses, the microcysts diagnosed in the domestic ducks examined in this study were named as a new species. This is the first record of *Sarcocystis* spp. from waterfowl in China. Sarcocysts of similar morphology occur frequently in different Anseriformes birds, and the relationships among these species need to be further clarified in future studies using more molecular markers.

**Graphical Abstract:**

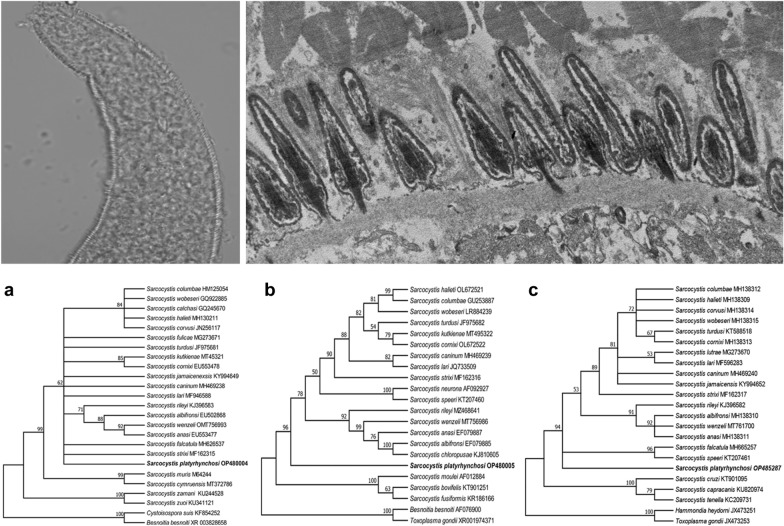

## Background

Species of the genus *Sarcocystis* are cyst-forming intracellular protozoan parasites with an obligate two-host life-cycle between predators (definitive hosts) and their prey animals (intermediate hosts). Current identification and classification of the *Sarcocystis* species in a given host mainly depend on the ultrastructure of the sarcocyst walls and the nucleotide sequences of a number of molecular markers [1].

Avian species within the order Anseriformes are intermediate hosts of four *Sarcocystis* species, *S*. *rileyi*, *S*. *wobeseri*, *S*. *anasi* and *S*. *albifronsi*. Among these, *S. rileyi* forms macrocysts that resemble a grain of rice, and the remaining three species form microcysts that are invisible to the naked eye [2–4]. Here, we report the morphological and molecular characterization of a new *Sarcocystis* species that forms microcysts in skeletal muscles of the domestic duck *Anas platyrhynchos* in China. We also investigated the phylogenetic relationships of the new species with other avian-infecting *Sarcocystis* spp. using three molecular markers, 18S ribosomal DNA (18S rDNA), 28S ribosomal DNA (28S rDNA) and mitochondrial cytochrome oxidase subunit 1 (mt*cox*1).

## Methods

### Morphological observation of sarcocysts in domestic ducks

Muscle samples were collected from the legs of 26 domestic ducks purchased from a rural market located in Shuangtu, Yunyang county, Chongqing municipality, China, in January 2021. These free-range ducks were raised by local farmers. Twenty 3-mm-long pieces were obtained from each muscle sample, and these were pressed and squeezed between two glass slides for observation of sarcocysts under a stereomicroscope. The sarcocysts were then isolated from the muscle fibers using dissection needles and used for light microscopy (LM), transmission electron microscopy (TEM) and DNA studies.

For TEM, a total of four sarcocysts isolated from two domestic ducks were fixed first in 2.5% glutaraldehyde in cacodylate buffer (0.1 M, pH 7.4) and then in 1.0% osmium tetroxide, followed by dehydration through an increasing concentration of ethanol and embedding in Durcupan. Ultrathin sections were stained first with uranyl acetate (35 mg/ml) and then with lead citrate (35 mg/ml), followed by examination under a JEM100-CX transmission electron microscope (JEOL Ltd., Tokyo, Japan) at 80 kV. For DNA extraction, individual cysts were stored in sterile water at − 20 °C prior to processing.

### DNA isolation, PCR amplification, cloning and sequence analysis

Three sarcocysts, each obtained from a different domestic duck, were separately subjected to genomic DNA extraction using a TIANamp Genomic DNA Kit (Tiangen Biotech Ltd., Beijing, China) according to the manufacturer’s instructions. The primer pairs used for amplification of the three genetic markers were as follows: 18S DSF (5ʹ-CGTGGAAGGGTAGTGTTTA-3ʹ) and 18S DSR (5ʹ-CGGAAACCTTGTTACGACT-3ʹ) for 18S rDNA [5]; KL1 (5ʹ-TACCCGCTGAACTTAAGC-3ʹ) and KL3 (5ʹ-CCACCAAGATCTGCACTAG-3ʹ) for 28S rDNA [5]; SF1 (5ʹ-ATGGCGTACAACAATCATAAAGAA-3ʹ) [6] and CODSR (5ʹ-CCTCTAATCCTACGGTCATC-3ʹ) for mt*cox*1. The primers 18S DSF, 18S DSR and CODSR used in the study were designed using OLIGO 7.60 primer analysis software (Molecular Biology Insights, Inc., Cascade, CO, USA) according to the highly conserved regions of 18S rDNA and mt*cox*1 sequences of *Sarcocystis* spp. from fowls that are available in GenBank.

PCR amplifications were performed as previously described [7]. The resulting PCR products were gel purified using an E.Z.N.A.® Gel Extraction Kit (Omega Bio-Tek, Inc., Norcross, GA, USA) and ligated to the pCE2 TA/Blunt-Zero vector using a 5 min TA/Blunt-Zero Cloning Kit (Vazyme Biotech Co., Ltd. Nanjing, China) according to the manufacturer’s instructions. The ligated vectors were transformed into Trelief® 5α Chemically Competent Cell (Tsingke Biotechnology Co., Ltd., Beijing, China). The positive bacterial clones were sequenced on both directions by an ABI PRISM TM 3730 XL DNA Analyzer (Applied Biosystems, Thermo Fisher Scientific, Waltham, MA, USA).

Phylogenetic analyses were conducted on the nucleotide sequences of the three loci using MEGA X software [8]. Maximum likelihood (ML) trees of the 18S rDNA, 28S rDNA and mt*cox*1 sequences were constructed with the Kimura 2-parameter, Hasegawa-Kishino-Yano and Hasegawa-Kishino-Yano models, respectively. The reliability of the ML phylograms was tested via the bootstrap method using 1000 replications.

## Results

### Observation of sarcocysts in domestic ducks by LM and TEM

**Fig. 1:**
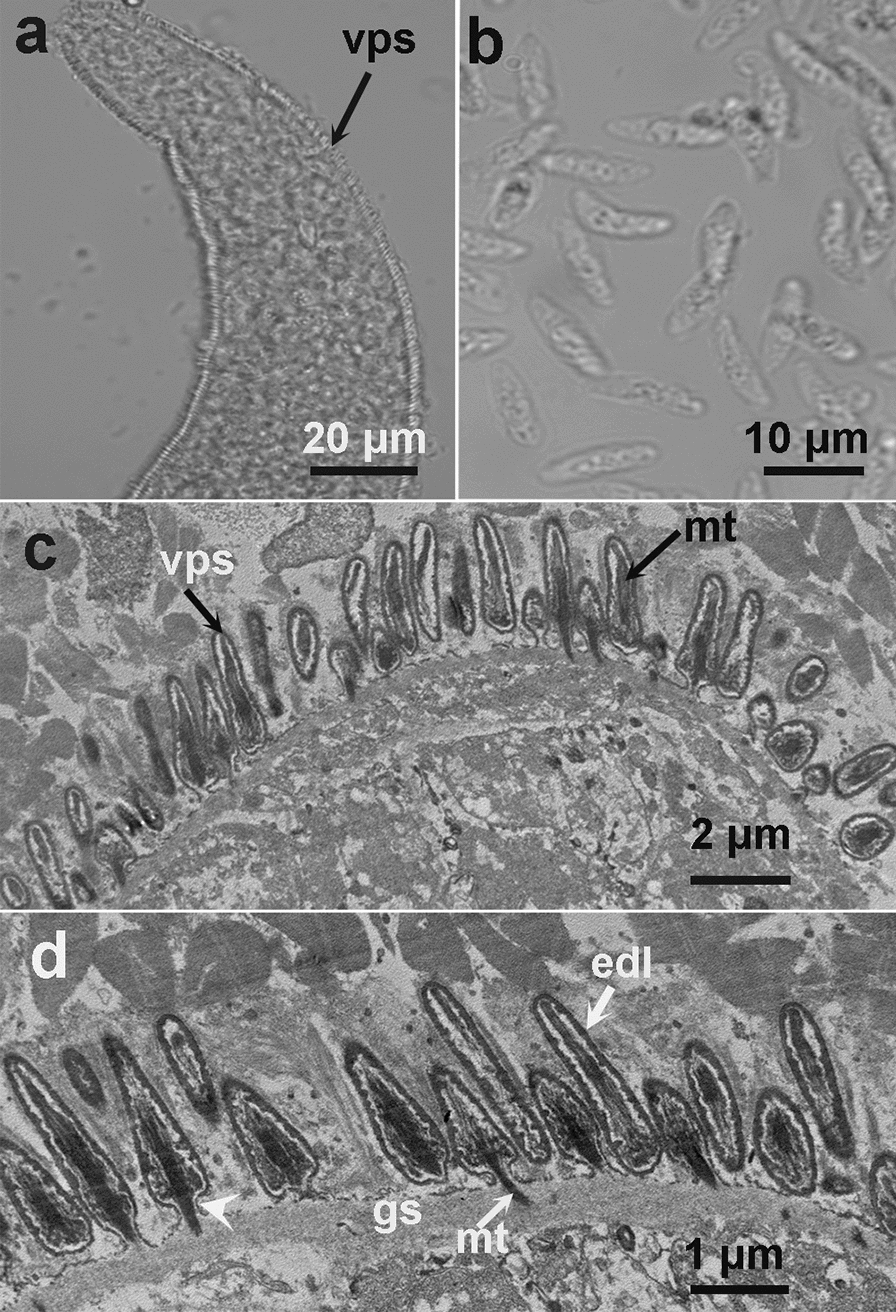
Morphological characteristics of *Sarcocystis platyrhynchosi* n. sp. isolated from the skeletal muscle of domestic ducks. **a** Light microscopy (LM) micrograph of a sarcocyst (unstained). Note the short brush-like villar protrusions (vps). **b** LM micrograph of lancet-like bradyzoites (unstained).** c** Transmission electron microscopy (TEM) micrograph of a sarcocyst. Note the lanceolated villar protrusions (*vps*) and the bundles of microtubes (*mt*) within the vps. **d** TEM micrograph of a sarcocyst. Note the narrowed stalk (arrowhead) of the vps, bundled mt extending into the ground substance (*gs*) and the smooth electron dense layer (*edl*) lining the vps

The LM study revealed the presence of sarcocysts in the leg muscle samples from three of the 28 domestic ducks studied (10.7%). The number of sarcocysts was relatively low, and only one to two sarcocysts were found in 20 g of muscle tissues from each of these three ducks. All sarcocysts were morphologically similar. The sarcocysts were microscopic, measuring 980–1694 × 72–140 μm (*n* = 8) in size and had a thick striated cyst wall with numerous brush-like villar protrusions (vps) of 3.8–4.3 μm in length (*n* = 30) on the cyst surface (Fig. 1a). They were septate and contained lancet-like bradyzoites of 12.3–14.0 × 1.6–2.3 μm (*n* = 30) in size (Fig. 1b).

The sarcocyst wall contained numerous lanceolated vps of 2.3–2.5 × 0.3–0.5 μm (*n* = 10) in size. Each vps narrowed in the stalk, widened in the middle, tapered at the end and was lined by a smooth electron-dense layer. The bundled microtubules were present in the core of the vps, which extended from tips of the vps into the ground substance. A layer of the ground substance of 0.3–0.5 μm in thickness (*n* = 8) was located immediately beneath the cyst wall (Fig. 1c, d).

### Molecular analyses of sarcocysts in domestic ducks

The three selected genetic markers (18S rDNA, 28S rDNA and mt*cox*1) were successfully amplified and sequenced. One nucleotide sequence was assembled from three clones of each genetic marker for each duck, and the sequences of each of the three genetic markers were determined to be 100% identical among the three ducks. Therefore, only one sequence of each genetic markers was deposited in GenBank under the accession numbers OP480004 for 18S rDNA (1594 bp), OP480005 for 28S rDNA (1558 bp) and OP485287 for mt*cox*1 (883 bp).

Comparisons of the new sequences with those deposited in GenBank showed that the most similar sequences were those of *Sarcocystis* *halieti* in the great cormorant *Phalacrocorax carbo* and European starling *Sturnus vulgaris*, and *Sarcocystis calchasi* in the domestic pigeon (*Columba livia*) at the 18S rDNA (99.1% identity); *Sarcocystis* *wenzeli* from the domestic chicken *Gallus gallus* at the 28S rDNA (95.9–96.0% identity); and *Sarcocystis speeri* from the opossum at the mt*cox*1 (98.2% identity). The new sequences shared no more than 99.0%, 95.6% and 97.7% identity at the 18S rDNA, 28S rDNA and mt*cox*1, respectively, with those of *Sarcocystis* *wobeseri*, *S. anasi, S*. *rileyi* and *S*. *albifronsi* obtained from Anseriformes avian species (Table 1).

**Table 1:** Similarities between the nucleotide sequences of *Sarcocystis platyrhynchosi* n. sp. with those of *Sarcocystis* spp. previously deposited in GenBank

Genetic markers	Accession numbers	Comparison between the newly obtained sequences (present study) with those previously deposited in GenBank		
		*Sarcocystis* species	Accession numbers	Similarity (%)
18S rDNA	OP480004	*S*. *halieti*	MH130211, JQ733511, MZ329690	99.1
		*S. calchasi*	GQ245670	99.1
		*S. wobeseri*	GQ922885, GQ922886, EU502869, HM159419	99.0
		*S. anasi*	EU553477	98.7
		*S*. *rileyi*	HM185742, MZ151434, KJ396583, GU120092	98.7
		*S*. *albifronsi*	EU502868	98.6
28S rDNA	OP480005	*S*. *wenzeli*	MT756986–MT756989	95.9–96.0 (average 96.0)
		*S. wobeseri*	GQ922887, GQ922888, HM159420, LR884239	94.3
		*S. anasi*	EF079887	95.3
		*S*. *rileyi*	MZ468641, MZ151434, GU188426, HM185743, KJ396585	95.4–95.6 (average 95.5)
		*S. albifronsi*	EF079885	94.9
*cox*1	OP485287	*S. speeri*	KT207461	98.2
		*S*. *falcatula*	MH665257	98.1
		*S. wobeseri*	MH138315	97.7
		*S. anasi*	MH138311	95.0
		*S. rileyi*	KT184389, KJ396582	96.5
		*S*. *albifronsi*	MH138310	95.2

*cox1* Cytochrome oxidase subunit 1,* rDNA* ribosomal DNA

### Phylogenetic analysis

**Fig. 2:**
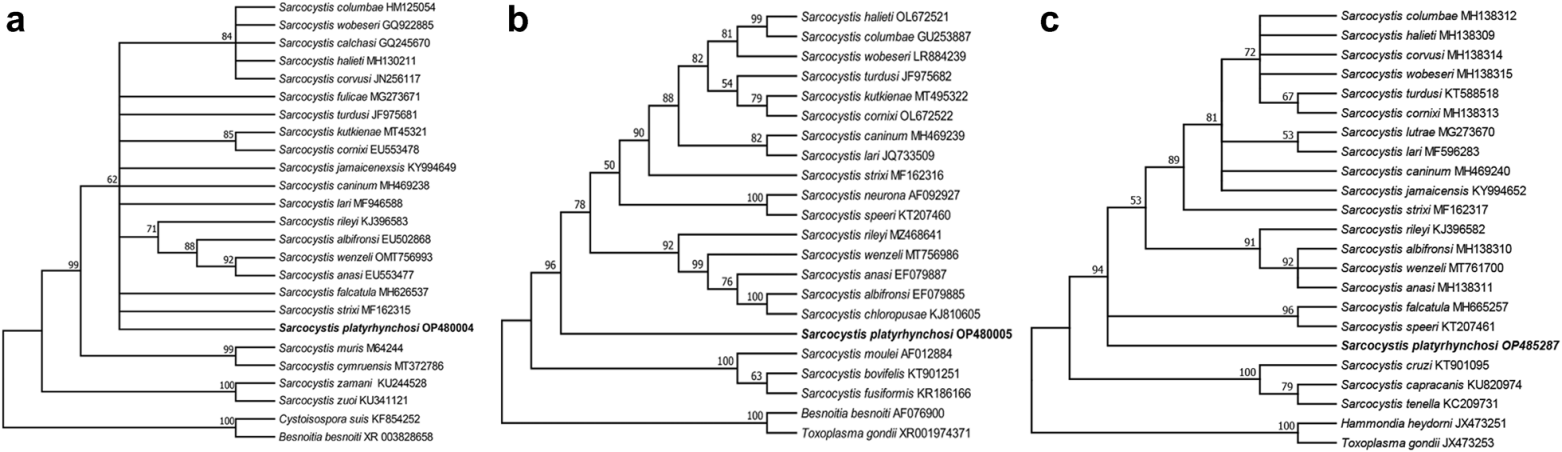
Phylogenetic trees of selected members of *Sarcocystis* species. The trees were conducted using 18S rDNA (**a**), 28S rDNA (**b**) and m*cox*1 (**c**) sequences using maximum likelihood (ML) with the Kimura 2–parameter, Hasegawa–Kishino–Yano and Hasegawa–Kishino–Yano models, respectively. The values between the branches represent bootstrap values per 1000 replicates. Values < 50% are not shown. *Besnoitia besnoiti*, *Cystoisopora suis*, *Toxoplasam gondii* or *Hammondia heydorni* were selected to root these trees. The newly obtained sequences of the 18S rDNA (OP480004), 28S rDNA (OP480005) and mt*cox*1 (OP485287) for *Sarcocystis platyrhynchosi* n. sp. are shown in bold. The phylogenetic trees inferred from the three genes had similar topologies, and *Sarocystis platyrhynchosi* formed a separate branch within a group encompassing *Sarcocystis* spp. obtained from avian or carnivorous intermediate hosts and avian marsupial, or carnivorous definitive hosts

Phylogenetic analysis obtained with the 18S rDNA (Fig. 2a), 28S rDNA (Fig. 2b) and mt*cox*1 (Fig. 2c) sequences placed the organism found in the domestic ducks within a clade encompassing *Sarcocystis* spp. obtained from avian or carnivorous intermediate hosts and avian, marsupial or carnivorous definitive hosts. All three phylogenetic trees had similar topologies, and the organism described in the present study formed a separate branch, which did not cluster with any of the three main clades of *Sarcocystis* species using birds as intermediate hosts, i.e. *S*. *calchasi*, *S*. *halieti* and others (bird–bird life-cycle), *S*. *rileyi*, *S*. *wenzeli* and others (birds–placental predatory mammals life-cycle), *S*. *falcatula*, *S*. *speeri* and others (birds–marsupial/specifically opossum life-cycle).

On the basis of morphological and molecular characterization of the sarcocysts, the isolate from the domestic ducks from China is regarded as a new species named *Sarcocystis platyrhynchosi* n. sp.

**Family Sarcocystidae Poche, 1913**

*Sarcocystis platyrhynchosi* n. sp.

*Diagnosis:* The sarcocysts were microscopic, up to 1694 μm long and 140 μm wide. Numerous brush–like vps of 3.8–4.3 μm in length were present on the cyst surface. TEM observation revealed that the sarcocysts had lanceolated vps of 2.3–2.5 × 0.3–0.5 μm, which narrowed in the stalk. Each vps contained bundled microtubules at the core that penetrated diagonally into the ground substance.

**Taxonomic summary**

*Type intermediate host*: Domestic duck *Anas platyrhynchos*.

*Type locality:* Shuangtu (31°15ʹ59ʺN, 108°94ʹ15ʺE, altitude 495 m a.s.l.), Yunyang County, Chongqing City, China.

*Site of infection:* Muscular tissues.

*Definitive host:* Unknown.

*Etymology:* Latin name of the intermediate hosts is used to name the species.

*Molecular characterization:* Sequences of the 18S rDNA (OP480004), 28S rDNA (OP480004) and mt*cox*1 (OP485287) of the new species have been deposited in GenBank. At the 28S rDNA and mt*cox*1 sequences, *S*. *platyrhynchosi* is unambiguously differentiated from *Sarcocystis* spp. obtained from Anseriformes birds.

*Deposited specimens:* Formalin–fixed tissues containing cysts of *S*. *platyrhynchosi*, as well as photomicrographs from LM and TEM examination of the sarcocysts, have been deposited at the Zoological Specimen Museum of Yunnan University, Kunming, China (collection number Prot202205).

*ZooBank registration:* To comply with the regulations set out in Article 8.5 of the amended 2012 version of the International Code of Zoological Nomenclature [9], details of the new species have been submitted to ZooBank. The Life Science Identifier (LSID) of the article is urn:lsid:zoobank.org:pub: 4FA38B3B-4505-4EF4-AC87-14067AD9FA7A. The LSID for the new species name *Sarcocystis platyrhynchosi* is urn:lsid:zoobank.org:act: D1663E66-23C2-4E50-96C5-9E336318C8EE.

#### Remarks

To date, only four named *Sarcocystis* species (*S*. *rileyi*, *S*. *wobeseri*, *S*. *anasi* and *S. albifronsi*) and two unnamed *Sarcocystis* species have been recorded in avian intermediate hosts of the order Anseriformes (Table 2). Among these, sarcocysts of *S*. *rileyi* are macroscopic, and those of the remaining five species are microscopic. The LM study revealed that the sarcocysts of *S*. *rileyi*, *S*. *wobeseri* and *Sarcocystis* sp. 2 ex *Anser caerulescens* have thin and smooth walls, and that those of *S. anasi*, *S. albifronsi* and *Sarcocystis* sp. 1 ex *A. caerulescens* have thick and striated walls characterized by radial spines- or finger-like vps on the cyst surface. The unltrastructures of the sarcocysts previously described from avian species of Anseriformes are categorized into three TEM wall types according to the classification provided by Dubey et al. [1]. One type includes vps with anastomosing branches that is similar to type 23 in *S*. *rileyi*; the second type has minute undulations on the cyst wall that is similar to type 1d for *S*. *wobeseri* and *Sarcocystis* sp. 2 ex *A. caerulescens*; and the third type has finger-like vps arranged in a palisade fashion that is similar to type 9a for *S*. *anasi*, *S. albifronsi* and *Sarcocystis* sp. 1 ex *A. caerulescens*. In our material, *S*. *platyrhynchosi* sarcocysts had short, brush–like vps on the cyst surface. The TEM study revealed that the lanceolated vps had a narrow stalk and contained bundled microtubules that penetrated diagonally into the ground substance; this is roughly similar to the TEM wall type 9d or 11a based on outlines of the lanceolated vps or the features of bundled microtubules extended into ground substance, respectively. This TEM wall type differs remarkably from those of *Sarcocystis* spp. obtained previously from wild mallard ducks and other species of Anseriformes.

**Table 2:** *Sarcocystis* spp. in the Anseriformes birds

*Sarcocystis* species	Intermediate host	Definitive host	Sarcocysts		Location	References
			Light microscopy study	Transmission electron microscopy study		
*S*. *rileyi* Stiles 1893	Shoveler duck *Anas clypeata*, mallard duck *Anas platyrhyncho*s, Eurasian wigeon *Anas penelope*, common teal *Anas crecca* and common eider *Somateria mollissima*	Skunk *Mephitis mephitis*), red fox *Vulpes vulpes* and raccoon dog *Nyctereutes procyonoides*	Macroscopic, up to 12 mm. Thick cyst wall (3–5 μm) without visible vps	Branched vps, type 23	Noth and South America and Europe	[2, 10–17]
*S*. *wobeseri* Kutkiené, Prakas, Sruoga, and Butkauskas 2010	Barnacle goose *Branta leucopsis*, mallard duck, herring gull *Larus argenticus*	Unknown	Microscopic, up to 6 mm long. Smooth, thin cyst wall (< 1 μm)	Conical vps, type 1d	Lithuania	[3, 18–20]
*S. anasi* Kutkiené, Prakas, Sruoga, and Butkauskas 2012	Mallard duck	Unknown	Microscopic, up to 5 mm long. Striated wall, vps up to 1.5 μm long	Palisade-like vps, type 9a	Lithuania	[4, 18]
*S. albifronsi* Prakas, Sruoga, and Butkauskas 2012	White-fronted goose *Anser albifons*	Arctic fox *Alopex legopus*	Microscopic, up to 4 mm long. Striated cyst wall, vps up to 2.4 μm long	Teat-or finger-like vps, type 9a	Lithuania	[4, 21]
*Sarcocystis* sp.1 Wober, Leighton, and Leighton 1981	Lesser snow goose *Anser caerulescens*	Unknown	Microscopic, up to 3.37 mm long. Thick cyst wall, radial spines-like vps, 1.5–2.0 μm long	Palisade-like vps, type 9a	Canada	[22]
*Sarcocystis* sp.2 Wober, Leighton, and Leighton 1981	Lesser snow goose	Unknown	Microscopic, 53.8 μm wide. Smooth, thin cyst wall, 0.5 μm thick	Invagination on the cyst wall, type 1d	Canada	[22]
*S*. *platyrhynchosi* n. sp.	Mallard duck	Unknown	Microscopic, up to 1649 μm long. Thick cyst wall, brush-like vps, up to 4.3 μm long	Lanceolated vps, bundled microtubules extended into ground substance	China	This study

*vps* villar protrusions

## Discussion

Sarcocysts have been found in the muscles of at least 20 avian species within the order Anseriformes in Europe and North and South America [2–4, 10–25]. In studies carried out during the previous century in birds in North America, macrocysts were observed in Anseriformes avian hosts by Riley in 1869, and all were subsequently designated as* S. rileyi* by Stiles in 1893 (see [2]). Microcysts were subsequently observed using LM in five named species of Anseriformes in North America [23]. These were classified into five cyst categories, namely 1a, 1b, 2a, 2b and 2c, based on the thickness of the cyst wall [23]. Microcysts were also found in 15 species of Anseriformes in Europe by LM, and these were divided into four microcysts types, I–IV [26]. Type I cyst found in 10 species had cyst walls up to 1.2 μm in thickness and no clear protrusions on the surface. The walls of type II cysts from four species were surrounded by palisade-like protrusions (up to 1.5 μm long) on the surface, and the type III cysts from four species had teat- or finger-like protrusions (up to 2.4 μm long) on the surface of the cyst walls. Type IV cysts found in two species had a wavy cyst wall. However, no species names for these microcysts were proposed by these authors at that time, mainly due to these sarcocysts being morphologically indistinguishable in different intermediate hosts.

Characterization of the ultrastructures of sarcocysts is a reliable method to identify and determine *Sarcocystis* species within a given host. The ultrastructure of cyst walls has been categorized into 42 types, with several subgroups [1]. The ultrastructures of macrocysts in the shoveler duck *Anas clypeata* [2] and mallard duck [10] were characterized for the first time in this century. The morphologically similar sarcocysts in these two hosts were designated as *S. rileyi* by the authors. Subsequently, mainly based on the ultrastructures of the microcysts, the type I cysts in the barnacle geese (*Branta leucopsis*) and type IV cysts in the mallard ducks were shown to be morphologically similar, resulting in them being named as one species, *S*. *wobeseri* [3]. However, the type II cysts ex mallard ducks and type III cysts ex white–fronted geese *Anser albifrons* were named *S*. *anasi* and *S. albifronsi* [4], respectively, even though they were also found to be morphologically similar, and classified into TEM wall type 9a [4, 18, 21]. In our materials, the ultrastructures of microcysts obtained from domestic ducks were roughly similar to the TEM wall type 9d or 11a, and were completely different from the those of *Sarcocystis* spp. reported in wild mallard ducks and wild geese.

Morphologically similar species occur frequently in avian species of Anseriformes, such as the macrocysts found in numerous duck species within the genera *Aix*, *Anas*, *Aythya*, *Bucephala* and *Melanitta* [2], leading to confusion as to whether they represent a same species of *Sarcocystis* in different intermediate hosts. In recent decades, molecular analysis has been used as a primary or supplemental tool to delineate or identify species of *Sarcocystis*, and different genetic markers have shown different levels of intra- or interspecific sequence diversity [6]. Based on nucleotide sequence analysis, all macrocysts found in the mallard duck, Eurasian wigeon *Anas penelope*, common teal *Anas crecca* and the common eider *Somateria mollissima* are considered to be *S*. *rileyi* [11, 12], and the intermediate hosts of *S*. *wobeseri* were revealed to be mallard ducks, barnacle geese and herring gulls *Larus argentatus* [3, 19, 20]. TEM wall type 9a sarcocysts in mallard ducks and white-fronted geese were specified as *S*. *anasi* and *S. albifronsi*, respectively, based on divergence of the internal transcribed spacer sequences (90.8% identity) and differences in the shapes and sizes of the bradyzoites [4]. Bradyzoites of 13.0–16.1 × 1.8–2.5 μm in *S. anasi* were slightly bent with blunt ends, widening toward one end [18], whereas those of *S. albifronsi* were almost straight and resembled a shuttle in shape, and measured 10.0–13.5 × 1.5–2.5 μm [21]. However, the two species could not be differentiated at the molecular level at the remaining four loci: 18S rDNA (99.7%), 28S rDNA (99.2%), *cox*1 (99.7%) and *rpo*B (99.3%) [19, 27]. Therefore, based on the limited data currently available, confusion remains regarding the relationship of the morphologically similar species of *Sarcocystis* in Anseriformes birds. In our materials, the 18S rDNA sequences of *S. platyrhynchosi* shared high similarity (≥ 99.0%) with those of *Sarcocystis* spp. in different avian species. This indicates that this gene is unsuitable for use as a marker to differentiate the closely related *Sarcocystis* spp. in avian species, confirming the findings reported in the closely related *Sarcocystis* spp. in domestic ruminants [6, 28]. However, the 28S rDNA and *cox*1 sequences of the new species could be unambiguously discriminated from those of *Sarcocsytis* spp. in different avian species reported in previous studies.

## Conclusions

To the best of our knowledge, this report is the first record of sarcocysts in waterfowls in China. To date, only limited data are available for *Sarcocystis* in wild ducks and geese, and studies of *Sarcocystis* in domestic ducks are especially rare. In the present study, the microcysts identified in the domestic ducks were named as a new species based on morphology and DNA sequence analyses, but the definitive host of the new species is still unknown. Morphologically similar sarcocysts occur frequently in different avian species of Anseriformes, and the relationships among these species need to be further clarified using more molecular markers in the future.

## Data Availability

The datasets used and/or analyzed during the current study are available from the corresponding author upon reasonable request. Nucleotide sequences of the 18S rDNA (OP480004), 28S rDNA (OP480005), and mitochondrial *cox*1 (OP485287) of the new species have been deposited in GenBank.

## Abbreviations

*cox*1: Cytochrome oxidase subunit 1
LM: Light microscopy
mt: Mitochondrial
18S rDNA: 18S ribosomal DNA
28S rDNA: 28S ribosomal DNA
TEM: Transmission electron microscopy
vps: Villar protrusions
